# Vector competence evaluation of mosquitoes for Tahyna virus PJ01 strain, a new *Orthobunyavirus* in China

**DOI:** 10.3389/fmicb.2023.1159835

**Published:** 2023-04-20

**Authors:** Tong Cai, Ran Liu, Yuting Jiang, Nan Jia, Xianyi Jian, Xiaolan Cheng, Fenglin Song, Xiaoxia Guo, Tongyan Zhao

**Affiliations:** ^1^State Key Laboratory of Pathogen and Biosecurity, Institute of Microbiology and Epidemiology, Beijing, China; ^2^Dalian International Travel Healthcare Center (Dalian Customs Port Clinic), Dalian, China

**Keywords:** Tahyna virus, *Orthobunyavirus*, vector competence, *Aedes albopictus*, *Culex pipiens pallens*

## Abstract

**Introduction:**

Tahyna virus (TAHV), an arbovirus of the genus *Orthobunyavirus*, is a cause of human diseases and less studied worldwide. In this study, a new strain of TAHV was isolated from *Aedes* sp. mosquitoes collected in Panjin city, Liaoning province. However, the competent vector of TAHV in China is still unknown.

**Methods:**

The genome of newly isolated TAHV was sequenced and phylogenetic analysis is performed. *Aedes albopictus* and *Culex pipiens pallens* were orally infected with artificial virus blood meals (1:1 of virus suspension and mouse blood), the virus was detected in the midgut, ovary, salivary gland and saliva of the mosquitoes. Then, the transmission and dissemination rates, vertical transmission and horizontal transmission of the virus by the mosquitoes were assessed.

**Results:**

Phylogenetic analysis revealed that the virus shared high similarity with TAHV and was named the TAHV PJ01 strain. After oral infection with virus blood meals, *Ae. albopictus* showed positive for the virus in all tested tissues with an extrinsic incubation period of 2 days and a fluctuating increasement of transmission and dissemination rates. Whereas no virus was detected in the saliva of *Cx. pipiens pallens*. Suckling mice bitten by infectious *Ae. albopictus* developed obvious neurological symptoms, including inactivity, hind-leg paralysis and difficulty turning over, when the virus titer reached 1.70×10^5^ PFU/mL in the brain. Moreover, TAHV was detected in the eggs, larvae and adults of F1 offspring of *Ae. albopictus*.

**Discussion:**

*Ae. albopictus* is an efficient vector to transmit TAHV but *Cx. pipiens pallens* is not. *Ae. albopictus* is also a reservoir host that transmits the virus vertically, which further increases the risk of outbreaks. This study has important epidemiological implications for the surveillance of pathogenic viruses in China and guiding comprehensive vector control strategies to counteract potential outbreaks in future.

## 1. Introduction

Genus *Orthobunyavirus*, belonging to the family *Peribunyaviridae* order *Bunyavirales*, is a spherical virion with a diameter of 70–110 nm and surrounded by a lipid envelope (Abudurexiti et al., 2019; Dias et al., 2022). Similar to other members of the family *Peribunyaviridae*, the genome of orthobunyaviruses consist of a single-stranded negative sense RNA, which is tri-segmented into S (small), M (medium) and L (large) segments, encoding proteins with different functions (Elliott, 2014). So far, more than 170 viruses of the genus *Orthobunyavirus* have been identified, and they are grouped into 18 serogroups based on their serological relatedness (Elliott, 2014). California serogroup (CSG) is one of the groups that can be transmitted to vertebrates through an arthropod vector and has been shown to negatively affect human health. Eight pathogenic CSG species, including the La Crosse virus (LACV), Jamestown Canyon virus (JCV), California encephalitis virus (CEV), Snowshoe hare virus (SSHV), Chatanga virus (CHATV), Inkoo virus (INKV), Keystone virus (KEYV), and Tahyna virus (TAHV), have been associated with neuroinvasive disease, febrile illness and/or diffuse rash in Asia, Africa, Europe and/or North America (Evans and Peterson, 2019). However, due to the lack of vaccine and targeted drugs, vector control is still the main way to reduce the risk of outbreaks of arboviruses-related diseases.

Mosquitoes are undoubtedly regarded as the most important arthropod vectors, many of which have the ability to carry and transmit a variety of pathogenic viruses belonging to genera *Orthobunyavirus*, *Flavivirus* and *Alphavirus* etc (Cheng et al., 2016). Their inherent ability to acquire, maintain and transmit pathogens is called vector competence, which is affected by multiple factors such as mosquito species, virus strain and the interaction/co-evolution between them, as well as other non-genetic and epigenetic factors (Houe et al., 2019). Although CSG viruses have variable vector preferences, primary vectors for several CSG viruses have been described, including *Aedes triseriatus* for LACV, *Aedes dorsalis* for CEV, *Aedes trivittatus* for Trivittatus virus (TVTV), *Aedes atlanticus* for KEYV, and *Aedes squamiger* for Morro Bay virus (MBV) (Evans and Peterson, 2019). However, existing studies have mainly focused on LACV and a few other CSG viruses, and there are few studies on TAHV. To date, TAHV has been detected worldwide in four genera, including *Aedes*, *Anopheles*, *Culex* and *Culiseta*, of which *Aedes vexans* and *Aedes cantans* are considered to be the primary vectors in Europe (Hubalek, 2008; Kilian et al., 2013; Hubalek et al., 2014). Nevertheless, differences in geographic location, TAHV evolution and dominant mosquito species are bound to cause differences in mosquito vectors across regions. In China, TAHV has only been isolated four times from genera *Aedes* and *Culex* (Gu and Artsob, 1987; Lu et al., 2009; Li et al., 2010; Lu et al., 2011), and to our best knowledge, the competent vector of TAHV is still unknown.

TAHV was first isolated in 1958 in the former Czechoslovakia and subsequently widely identified in central Europe (Bardos and Danielova, 1959). In China, TAHV was first isolated in 2006 from *Culex* sp. mosquitoes collected in Xinjiang Uygur Autonomous Region (Lu et al., 2009). In 2007 and 2008, different geographical strains of TAHV were successively isolated in Xinjiang Uygur autonomous region, Qinghai province and Inner Mongolia autonomous region (Li et al., 2010; Cao et al., 2011; Lu et al., 2011). In this study, TAHV was refound in Panjin city, Liaoning province in 2019, which is the first report of TAHV isolation in eastern coastal areas of China, suggesting the propensity of TAHV to spread from west to east in China and its potential to cause large-scale epidemics. In Europe, TAHV has been shown to cause a flu-like illness called “Valtice fever” though rarely a neuroinvasive disease (Sluka and Simkova, 1972); And in China, TAHV was suspected as a possible etiologic agent of febrile illness with pulmonary involvement and central nervous system disease (Lu et al., 2009). However, diseases caused by CSG viruses are likely to be underreported because of the difficulty of diagnosis: only a very small number of CSG viruses have been characterized, and patients who do not seek treatment and those with less severe disease might not be included in case reports because patients are not routinely tested for CSG viruses unless arbovirus disease is suspected.

Genera *Aedes* and *Culex* are the most common anthropophilic mosquito genera in China. *Aedes albopictus*, the dominant *Aedes* sp. in China, is one of the fastest spreading and most threatening invasive species in the world, which have spread from their origin in Southeast Asia to every continent except Antarctica in the past 20 years (Kraemer et al., 2015). *Culex pipiens pallens*, the dominant *Culex* sp. in North and Northeast China, is widely distributed in nearly one-third of China. Both have been shown to be vectors of a variety of mosquito-borne viruses, such as Zika Virus (ZIKV), Dengue virus (DENV), Western equine encephalitis virus (WEEV) and West Nile virus (WNV) (Wang et al., 2010; Guo et al., 2013; Jiang et al., 2014; Guo et al., 2020). With global warming and the increase of human-assisted spread, habitats of mosquitoes in China have a trend of large-scale expansion, which poses a great threat to public health. Therefore, the monitoring and control of mosquitoes and the viruses they carry and transmit are increasingly important.

This study describes a novel member of the genus *Orthobunyavirus*, tentatively named TAHV PJ01 strain, which was isolated from *Aedes* sp. mosquitoes collected from Panjin city, Liaoning province, China (GPS location: 41°12′ N and 122°07′ E). Here we explore the phylogenetic analysis of TAHV PJ01 strain, and comprehensively evaluate for the first time the vector competence of two dominant mosquitoes in China against it, including replication potential, horizontal transmission and vertical transmission. This study enriched the species diversity of the genus *Orthobunyavirus* in China, hinting at the potential emerging infectious diseases and their transmission routes, which is of great epidemiological significance for the development of comprehensive vector control strategies for the prevention of arboviral diseases.

## 2. Materials and methods

### 2.1. Mosquito strains

Two mosquito strains were used in this study. *Ae. albopictus* Guangzhou strain was originally collected as larvae from Guangzhou city, Guangdong Province (GPS location: 23°07′ N and 113°16′ E), in 2019. *Cx. pipiens pallens* Beijing strain was originally collected as larvae from Beijing city (GPS location: 39°28′ N and 116°28′ E), in 2013. Both mosquito strains were reared under standard insectary conditions at 26 ± 1°C and 75 ± 5% relative humidity (RH) with a photoperiod of 14 h light: 10 h dark cycles. Prior to the infectious feed, adult mosquitoes were provided with 8% sucrose solution.

### 2.2. Cell lines, virus stocks, and animals

C6/36 (*Ae. albopictus*) and BHK-21 (baby hamster kidney) cells were maintained in our laboratory. C6/36 cells were cultured in RPMI 1640 medium (Gibco) supplemented with 10% fetal bovine serum (FBS) (Gibco) and 1% penicillin/streptomycin (Gibco) at 28°C in an incubator of 5% CO_2_. BHK-21 cells were cultured in Dulbecco’s modified Eagle medium (DMEM) (Gibco) supplemented with 10% FBS and 1% penicillin/streptomycin at 37°C in an incubator of 5% CO_2_.

The TAHV PJ01 strain isolate was originally isolated from *Aedes* sp. mosquitoes collected in Panjin city, Liaoning province (GPS location: 41°12′ N and 122°07′ E) in 2016, and used as a seed stock by passaging once in C6/36 cells and twice in BHK-21 cells. Then, the working stock was generated by inoculation into C6/36 cells, which was subsequently harvested and stored as individual 1.5 mL aliquots in freezing tubes at −80°C. The virus titers were 1.15 × 10^7^ plaque-forming unit (PFU)/mL as detected by plaque assay.

25–35 g female Kunming mice were used as a blood source and 4-day-old suckling Kunming mice were used as viral infection animal models in this study. All Kunming mice were purchased from Beijing VITAL RIVER Laboratory Animal Technology Co., LTD., and transferred to our laboratory and acclimated to the new environment for 24 h prior to the experiment to minimize the effects of transport.

### 2.3. Viral genome sequencing

Viral RNA of TAHV PJ01 strain was firstly extracted from virus stock by QIAamp Viral RNA Mini Kit (QIAGEN), and then reverse transcribed into cDNA using Eastep® RT Master Mix Kit (Promega) according to manufacturer’s instructions. The genome of TAHV consists of 3 segments: L, M and S. The middle region and the ends of segments were amplified by PCR and RACE (Rapid amplification of cDNA ends), respectively. The following reagents were used for PCR reactions: 2 μL of cDNA, 1 μL of forward primer, 1 μL of reverse primer, 10 μL of PrimeSTAR Buffer, 4 μL of dNTP Mixture, 0.5 μL of PrimeSTAR HS DNA Polymerase, and 31.5 μL of nuclease-free water to yield a 50 μL final reaction volume. Amplification reactions were performed in a PCR system (Biometra Tadvanced) programmed as follows: 30 cycles at 98°C for 10 s, 55°C for 5 s and 72°C for 60 s. To obtain the 3′ and 5′ ends sequence, a polyadenylated tail was added to the RNA genome by Poly(A) Polymerase (TaKaRa). Then, first-strand cDNA synthesis, RACE and In-Fusion cloning of RACE products were performed using SMARTer® RACE 5′/3’ Kit (TaKaRa) according to user manual. PCR and RACE products were sequenced at Shenzhen BGI Co., LTD (Shenzhen, China). Genome assembly was performed using SnapGene 2.3.2 (Insightful Science, USA). All primers used for amplification were listed in Supplementary Table 1.

### 2.4. Phylogenetic analyses

The complete sequences of all three segments of 22 strains (except the MBV, which had only S segment sequence in the GenBank) belonging to the CSG in the genus *Orthobunyavirus* together with the representative members of the Bunyamwera serogroup (Bunyamwera virus) were used in the analysis (from GenBank as of 6 June 2022). Accession numbers of all the above strains are listed in Supplementary Table 2. Alignment of sequences was conducted by the ClustalW function in MEGA-X (Kumar et al., 2018). Phylogenetic analysis among nucleotide sequences was constructed by Neighbor-Joining method together with 1,000 replications bootstrap using MEGA-X. Pairwise distances were analyzed using SDTv1.2 (Muhire et al., 2014) and visualized by R package corrplot.

### 2.5. Oral infection of mosquitoes

Virus-infected blood meals were prepared containing 1:1 mouse blood and TAHV PJ01 strain suspension supplemented with 3% FBS and 1% heparin sodium. Seven-day-old adult female mosquitoes that had been starved for 12 h were fed with this infected blood meal using a Hemotek membrane feeding system for *Ae. albopictus* and a saturated sponge for *Cx. pipiens pallens*. The infected blood meal was kept at 37°C during feeding. After 50 min of feeding, mosquitoes were cold-anesthetized, and blood-engorged mosquitoes were transferred and maintained in the standard conditions.

### 2.6. Mosquitoes processing

To evaluate the infection of the TAHV PJ01 strain in *Ae. albopictus* and *Cx. pipiens pallens*, 30 blood-engorged mosquitoes were, respectively, sampled at 2, 4, 6, 8, 10, 12, 16 and 18 day post exposure (dpe). The midguts, ovaries, salivary glands, and saliva of all sampled mosquitoes were dissected and collected carefully with sterile dissecting needles and individually transferred into 1.5-mL microtubes containing 1 mL of RNAiso Plus (TaKaRa). Then, organs were homogenized using 2.5 mm stainless steel grinding balls and an N▪9524R frozen grinder manufactured by Beijing HODER Technology Co., LTD. These samples were then used for RNA extraction and TAHV PJ01 strain detection *via* RT-qPCR.

### 2.7. Horizontal transmission experiments

To determine the ability of *Ae. albopictus* and *Cx. pipiens pallens* to horizontally transmit TAHV PJ01 strain, at 10 dpe, whole nests of 4-day-old suckling mice were placed in cages containing 50 mosquitoes that previously were fed with the virus-infected blood meals described above. 2 h after exposure, all the mosquitoes were removed from the cage and the whole bodies of newly blood-engorged mosquitoes were detected for virus infection by RT-qPCR. The suckling mice were also removed from the cage, counted red dots of mosquito bites and reared with their mother mouse. The suckling mice were then monitored daily. The healthy mice were sampled on days 4, 7 and 10 (2 mice *per* day) as routine sampling. The unhealthy mice were sampled immediately once found be sick or dead as additional sampling. For all of the above sampled mice, half of the brain was grinded in the DMEM and filtered to obtain supernatant for plaque assay to determine virus titer; The remaining portion of the brain was used for RNA extraction and TAHV PJ01 strain detection *via* RT-qPCR.

### 2.8. Transovarial transmission experiments

To determine the ability of *Ae. albopictus* to transovarial transmit TAHV PJ01 strain from parents to offspring, the eggs laid by infected mosquitoes during the first and second gonotrophic cycles were collected. In the first gonotrophic cycle, blood-engorged mosquitoes were allowed to oviposit at 6–10 dpe. The eggs were maintained in a desiccator at 26 ± 1°C and 90% RH with a photoperiod of 14 h light:10 h dark cycles for 7 days to permit embryonation, and were subsequently partially hatched. Eggs, hatched larvae, and emerged adults were tested for the presence of TAHV PJ01 strain by RT-qPCR. Then, parent female mosquitoes were fed with normal mouse blood at 10 dpe, and eggs from the second gonotrophic cycle were collected at 13–17 dpe. The eggs, larvae and adults of the second gonotrophic cycle were collected as above for TAHV PJ01 strain detection.

### 2.9. Virus detection

Total RNA from midguts, ovaries, salivary glands, saliva, eggs, hatched larvae and emerged adults was isolated using RNAiso Plus (TaKaRa), and from suckling mouse brain using RNeasy® Mini Kit (QIAGEN), according to the manufacturer’s instructions.

TAHV PJ01 strain was detected in these samples using GoTaq® Probe 1-Step RT-qPCR System (Promega). A pair of universal primers for CSG virus amplification (Lambert and Lanciotti, 2009) and a newly designed probe were used for detection and were listed in Supplementary Table 1. The following reagents were used for RT-qPCR reactions: 2 μL of RNA sample, 5 μL of GoTaq® Probe qPCR Master Mix, 0.2 μL of GoScript™ RT Mix, 0.5 μL forward primer, 0.5 μL reverse primer, 0.5 μL probe and 1.3 μL of nuclease-free water to yield a 10 μL final reaction volume. Amplification reactions were performed in a RT-qPCR system from LightCycler® 480 II (Roche) programmed as follows: 1 cycle at 45°C for 15 min, 95°C for 2 min, 40 cycles at 95°C for 15 s and 60°C for 30 s. Virus RNA (vRNA) copies were calculated by generating a standard curve using a recombinant plasmid containing virus segment insertion.

Virus titers in mouse brains were detected by plaque assay. The supernatants derived from the mouse brain virus suspensions were serially diluted and inoculated into BHK-21 cells cultured in a 6-well plate with 200 μL volumes. After 1 h incubation at 37°C of 5% CO_2_, virus stocks were discarded and the cells were overlaid with 1:1 of 2% ultra-low gelling agarose (SIGMA) and high sugar-DMEM (2×) (GENOM). Cells were incubated at 37°C of 5% CO_2_ and observed daily for cytopathic effect (CPE), after CPE emergence they were stained with a 2% crystal violet solution (2% crystal violet in 20% ethanol). The number of characteristic plaques was counted to represent the presence of the virus particles. The virus titers were expressed as PFU/mL.

### 2.10. Vector competence analysis

The vector competence of *Ae. albopictus* and *Cx. pipiens pallens* mosquito populations were characterized *via* the following indicators: positive rates and vRNA copies of midguts, ovaries, salivary glands and saliva; Infection rate (IR), transmission rate (TR), dissemination rate (DR) and dissemination efficiency (DE); Infection and morbidity of suckling mice; Infection of offspring in the first and second gonotrophic cycle.

### 2.11. Statistical analysis

GraphPad Prism 8.0 (GraphPad Software, San Diego, CA, USA) was used for data processing and visualization. SPSS 26 software (IBM, Armonk, NY, USA) was used for statistical analysis. Fisher’s exact test was used to compare rates. The Least Significant Difference (LSD) and the Tamhane test were used to compare the means ± standard errors (SEs) of RNA copy across multiple groups when passing and failing the homogeneity of variance test, respectively. An independent sample t-test was used to compare the means ± SEs of RNA copy between the two groups. *p* < 0.05 was considered statistically significant.

## 3. Results

### 3.1. Phylogenetic analysis of TAHV PJ01 strain

The virus was originally isolated from a pool of *Aedes* sp. mosquitoes and its genome was sequenced *via* the next-generation sequencing technology. The complete sequences of the virus have been submitted to the GenBank database (Accession No.: Segments S: OP727994.1; Segments M: OP727995.1; Segments L: OP727996.1). Analysis of the assembled sequences *via* the BLAST tool revealed that the newly isolated virus was the most correlated to TAHV, which belongs to the CSG of the genus *Orthobunyavirus*, and it was subsequently named TAHV PJ01 strain in this study.

**Figure 1:**
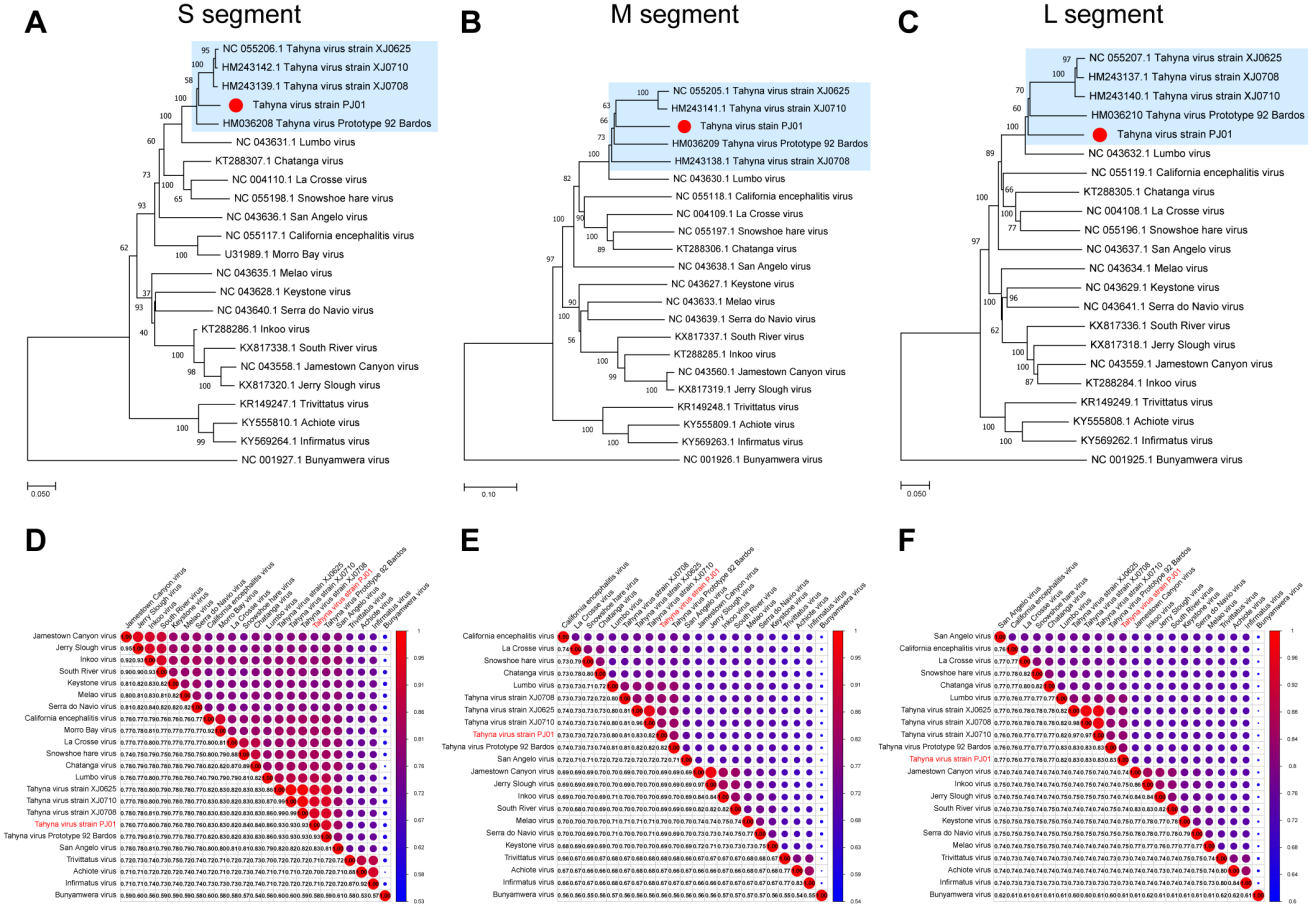
Neighbor-Joining phylogenetic trees **(A–C)** and pairwise distance **(D–F)** based on nucleotide sequences alignments of CSG of genus *Orthobunyavirus*. The Bunyamwera virus was used as an outgroup in the NJ trees. The scale bar indicates the evolutionary distance in the number of substitutions *per* nucleotide substitution/site, and the principal bootstrap support levels are indicated.

Together with the other members in the CSG of the genus *Orthobunyavirus*, the TAHV PJ01 strain was analyzed the phylogenetic relationship between the CSG based on all three segments, except the MBV, which only had complete CDSs for the S segments in GenBank. Bunyamwera virus, belonging to the Bunyamwera serogroup was used as the outgroup (Supplementary Table 2). The phylogenetic results of all three segments showed that the TAHV PJ01 strain belongs to the genus *Orthobunyavirus* and clusters with the members of CSG (Figures 1A–C). The overall topology of the phylogenetic trees obtained from the S and L segment sequences were similar, as the TAHV PJ01 strain was independent of the clade of TAHV isolated from Xinjiang Uygur Autonomous Region (highlighted in a light blue color in Figures 1A,C). However, for the M segment, the TAHV PJ01 strain was localized within the above clade (highlighted in a light blue color in Figure 1B).

The nucleotide identity analysis matrix of the selected viruses indicated the highest identity of the S segment whereas the lowest identity of the M segment in the CSG. The S, M, and L segments of TAHV PJ01 strain shared the highest sequence similarity with those of TAHV strain XJ0625 and XJ0710 (93.40%), TAHV strain XJ0625 (82.50%), and TAHV strain XJ0708 and XJ0710 (82.90%), respectively (Figures 1D–F).

### 3.2. Replication potential of TAHV PJ01 strain in *Ae. albopictus* and *Cx. pipiens pallens*

**Figure 2:**
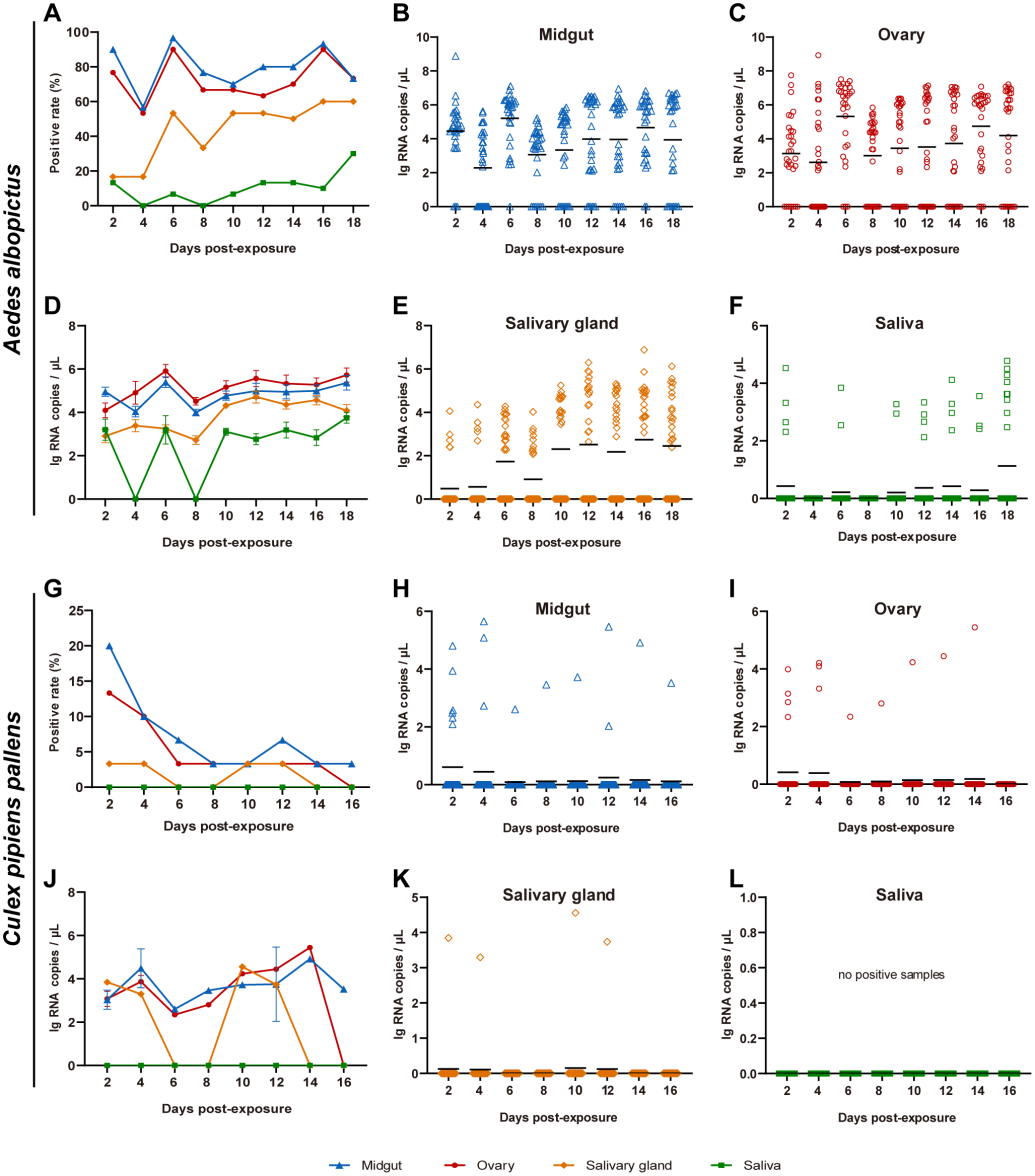
Replication potential of TAHV PJ01 strain in *Ae. albopictus* **(A–F)** and *Cx. pipiens pallens* **(G–L)**. *Ae. albopictus* and *Cx. pipiens pallens* were fed artificial virus blood meals containing of TAHV PJ01 strain suspension (50% v/v) and mouse blood (50% v/v). The midguts, ovaries, salivary glands, and saliva were dissected and collected for virus detection at the indicated days (*n* = 30). **(A,G)** Virus positive rate in *Ae. albopictus* and *Cx. pipiens pallens* (*n* = 30). **(B–F,H–L)** Viral RNA copy concentration in *Ae. albopictus* and *Cx. pipiens pallens*. In panels **(B,C,E,F,H,I,K,L)**, the black lines represented the means of all 30 samples. In panels **(D,J)**, data represented the means ± standard error of positive samples.

Virus replication in the midguts, ovaries, salivary glands, and saliva of *Ae. albopictus* and *Cx. pipiens pallens* were, respectively, tested at 2, 4, 6, 8, 10, 12, 16 and 18 dpe. For *Ae. albopictus*, from 2 dpe onwards, TAHV PJ01 strain could be detected in the midguts, ovaries and salivary glands on every sampling day (Figures 2A–E), and could be detected in saliva on all sampling days except 4 and 8 dpe (Figures 2A,D,F). Positive rate of midguts and ovaries remained high throughout the sampling days and peaked at 96.7 and 90.0% at 6 dpe and 6/16 dpe, respectively. Positive rate of salivary glands and saliva were generally maintained in a fluctuating upward trend, reaching 60.0 and 30.0% at 16/18 dpe and 18 dpe (Figure 2A). The vRNA copies of positive samples were calculated and showed a pattern of: ovary > midgut > salivary gland > saliva, with a maximum average vRNA copy of 5.91 ± 0.30 lg RNA copies/μL at 6 dpe, 5.40 ± 0.25 lg RNA copies/μL at 6 dpe, 4.71 ± 0.28 lg RNA copies/μL at 12 dpe and 3.75 ± 0.25 lg RNA copies/μL at 18 dpe, respectively (Figures 2B–F).

Compared with *Ae. albopictus*, *Cx. pipiens pallens* showed significant differences in TAHV PJ01 strain infection, with general downward trends of virus positive rates and all of them were less than 20.0% (Figure 2G). No virus was present in saliva on all sampling days, and by 16 dpe, no sample was virus-positive except 3.3% of the midguts (Figure 2G). Maximum vRNA copy of the few infected mosquitoes was 5.44 lg RNA copies/μL in ovaries at 14 dpe, 4.92 lg RNA copies/μL in midguts at 14 dpe, and 4.56 lg RNA copies/μL in salivary glands at 10 dpe (Figures 2H–L). The IR, TR, DR and DE of *Ae. albopictus* and *Cx. pipiens pallens* were also evaluated and were shown in Supplementary Table 3.

### 3.3. Horizontal transmission of TAHV PJ01 strain in *Ae. albopictus* and *Cx. pipiens pallens*

Horizontal transmission experiments of the TAHV PJ01 strain from mosquitoes to mice were performed. Positive results of virus detection in the mice brain means that the virus breaks through the blood–brain barrier to replicate in the suckling mice brain. In this study, more than one red blotch was present on the skin of all tested suckling mice after removal from the cage, which indicated that all tested suckling mice were bitten by at least one mosquito. Among the 50 mosquitoes used in each group, 37 *Ae. albopictus* were blood-engorged and positive with a vRNA copy of 6.11 ± 0.30 lg RNA copies/μL, 35 *Cx. pipiens pallens* were blood-engorged and 29 of them were positive with vRNA copy of 3.00 ± 0.24 lg RNA copies/μL.

**Figure 3:**
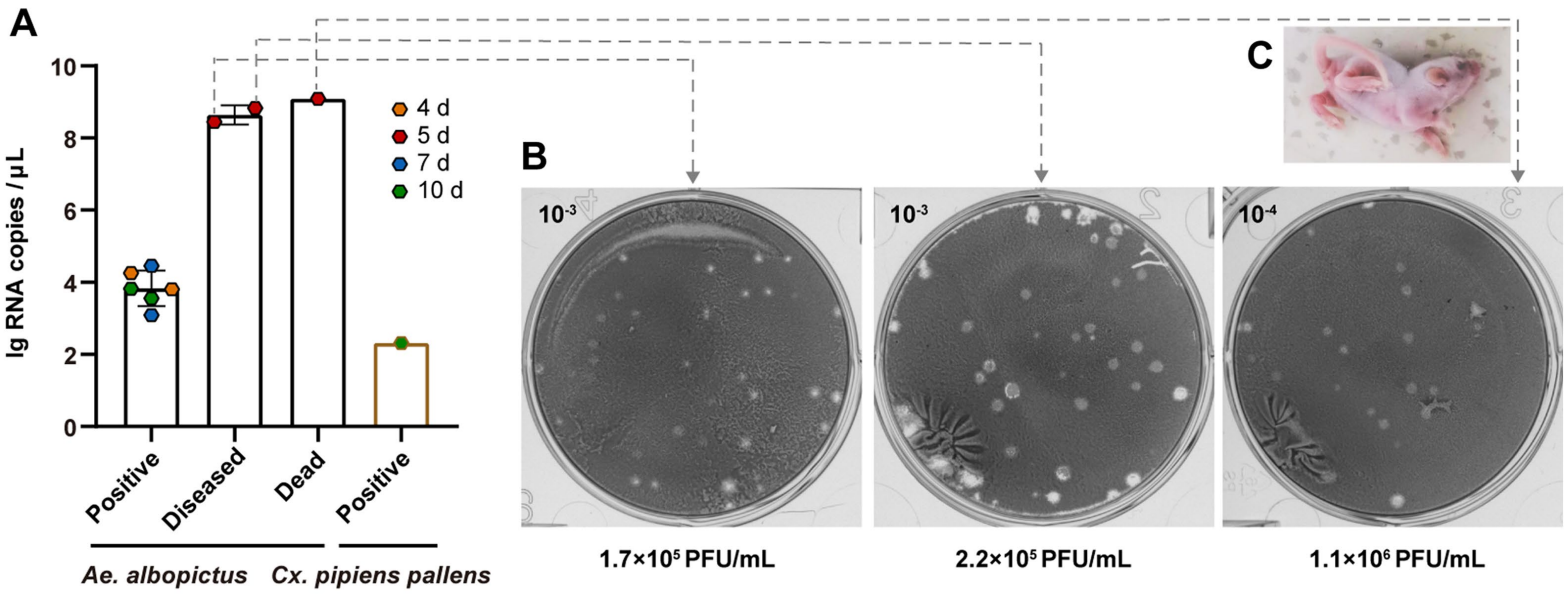
Assessment of horizontal transmission of TAHV PJ01 strain from *Ae. albopictus* and *Cx. pipiens pallens* to suckling mice. **(A)** RNA copies and **(B)** virus titers of TAHV PJ01 strain in suckling mice. **(C)** Physical characteristics of the diseased suckling mice.

There are significant differences between the two mosquito species in their ability to horizontally transmit TAHV PJ01 strain. Among 11 sucking mice that were bitten by *Ae. albopictus*, three (27.3%) developed obvious neurological symptoms, including inactivity, hind-leg paralysis and difficulty turning over, at day 5 (Figure 3C). One of these three (33.3%) diseased mice died within 24 h. Those 3 mice were sampled on day 5 and the other 8 were routinely sampled on days 4, 7 and 10 post-bite (2 mice *per* day and 2 additional on day 10). vRNA copies and virus titer in the brain of all the above mice were, respectively, detected *via* RT-qPCR and plaque assay. Results showed that nine mice, including those 2 diseased and 1 dead, 9 of the eleven (81.8%) suckling mice had vRNA in their brains within 10 days of observation (Figure 3A). Six viral RNA positive mice that exhibited no symptoms had an average vRNA copies of 3.83 ± 0.20 lg RNA copies/μL and no live virus, however there was no significant difference in the average vRNA copy among the different sampling days (*p* = 0.634). Two diseased mice had an average vRNA copies of 8.64 ± 0.19 lg RNA copies/μL, and their virus titers were 1.70 × 10^5^ PFU/mL and 2.20 × 10^5^ PFU/mL, respectively. The dead mouse had the highest vRNA copies of 9.09 lg RNA copies/μL accompanied by the highest virus titers of 1.10 × 10^6^ PFU/mL (Figures 3A,B). However, after being bitten by *Cx. pipiens pallens*, only one suckling mouse (12.5%) routinely sampled on day 10 had vRNA in its brain at a copy concentration of 2.32 lg RNA copies/μL (Figure 3A). No live virus was present and no illness or death were observed in this mouse within 10 days of observation.

### 3.4. Vertical transmission of TAHV PJ01 strain in *Ae. albopictus*

As *Ae. albopictus* exhibited more susceptibility to the TAHV than *Cx. pipiens pallens*, offspring of the infected *Ae. albopictus* mosquitoes in the different development stages (eggs, larvae and adults) were detected to demonstrate vertical transmission. A total of 1,500 F1 progeny in 90 pools in the first gonotrophic cycle and 750 F1 progeny in 48 pools in the second gonotrophic cycle were tested (Table 1). In the first gonotrophic cycle, the IR of eggs was 96.7% with a vRNA copy of 3.12 ± 0.17 lg RNA copies/μL. From the egg stage to the larvae stage, both IR and vRNA copies decreased significantly (*P*<0.001), reaching 30.0% and 1.52 ± 0.05 lg RNA copies/μL, and then steadily decreased to 13.3% and 1.48 ± 0.03 lg RNA copies/μL at the adult stage. In the second gonotrophic cycle, IR, MIR (minimum infection rate) and RNA copy all showed decreasing trends from the egg to the adult stage similar to those in the first gonotrophic cycle. At the adult stage, the IR was 13.3%, the MIR was 1.3%, and the vRNA copy was 1.43 ± 0.04 lg RNA copies/μL. However, there were no significant differences in the same stage between different gonotrophic cycles, except that vRNA copies of positive eggs and larvae in the second gonotrophic cycle were significantly higher than those in the first gonotrophic cycle (*P*<0.01). The fact that the TAHV PJ01 strain can be detected in adults of F1 progeny demonstrated that the TAHV PJ01 strain can be vertically transmitted from parents to offspring in *Ae. albopictus*.

**Table 1:** TAHV PJ01 strain infection of F1 progeny of *Aedes albopictus* in the first and second gonotrophic cycle.

Gonotrophic cycle	Stage	IR (%)	MIR (%)	RNA copy (lg/μL)
I	Egg (30/pool)	96.7 (29/30) ^aA^	3.2 (29/900) ^aA^	3.12 ± 0.17 ^aB^
	Larvae (10/pool)	30.0 (9/30) ^bA^	3.0 (9/300) ^aA^	1.52 ± 0.05 ^bB^
	Adult (10/pool)	13.3 (4/30) ^bA^	1.3 (4/300) ^aA^	1.48 ± 0.03 ^bA^
II	Egg (25/pool)	77.8 (14/18) ^aA^	3.1 (14/450) ^aA^	4.32 ± 0.35 ^aA^
	Larvae (10/pool)	20.0 (3/15) ^bA^	2.0 (3/150) ^aA^	1.82 ± 0.06 ^bA^
	Adult (10/pool)	13.3 (2/15) ^bA^	1.3 (2/150) ^aA^	1.43 ± 0.04 ^bA^

IR (Infection rate) = number of TAHV PJ01 strain-positive pools/total number of pools tested.

MIR (Minimum infection rate) = number of TAHV PJ01 strain-positive pools/total number of individuals (eggs, larvae, and adults) tested.

RNA copies are shown as the means ± standard errors (SEs).

The different lowercase letters indicate significant differences between different stages of the same gonotrophic cycle [IR and MIR: Fisher’s exact test, *p* < 0.05; RNA copy: Least Significant Difference (LSD) test, *p* < 0.05]. The different uppercase letters indicate significant differences between different gonotrophic cycles at the same stage (IR and MIR: Fisher’s exact test, *p* < 0.05; RNA copy: Independent sample *t*-test, *p* < 0.05).

## 4. Discussion

The present study characterized the TAHV PJ01 strain, which was previously isolated from *Aedes* sp. mosquitoes collected from Panjin city, Liaoning province, China (GPS location: 41°12′ N and 122°07′ E). The TAHV PJ01 strain was identified as most closely related to TAHV, belonging to Order *Bunyavirales*, family *Peribunyaviridae*, genus *Orthobunyavirus*, CSG. Several kinds of CSG viruses have been shown to cause disease in humans, ranging from mild rashes to severe neuroinvasive diseases, with symptoms including headache, fever, dizziness, vomiting, muscle weakness, stiff neck, seizures, hemiparesis, encephalitis, meningoencephalitis, and meningitis (Thompson et al., 1965; Fauvel et al., 1980; Gaensbauer et al., 2014; Drebot, 2015; Teleron et al., 2016; Lednicky et al., 2019), and some causing long-term and serious sequelae such as cognitive deficits and behavior changes (Huang et al., 1999; Byrd et al., 2018). Here, we described a novel member of CSG and characterized the vector competence of two common mosquito species in China to it, which was beneficial to understand the diversity of *Orthobunyavirus* in China and provide guidance for the prevention and control of arbovirus diseases caused by CSG virus.

In contrast to dengue virus, Japanese encephalitis virus and other mosquito-borne flaviviruses, orthobunyaviruses are quite underestimated with regard to their potential prevalence and harm. Panjin city is one of the important gateways of the China-Mongolia-Russia Economic Corridor and the 21st century Maritime Silk Road. It is located on the northeast coast of China and has the closest port to northeast China and eastern Inner Mongolia. As a result, Panjin city developed high-frequency exchanges and trade at home and abroad. In turn, the risk of large-scale epidemics and rapid transmission of vectors along with cargo transport has increased. Most importantly, Panjin city is rich in wetland resources, with a total area of 2,496 square kilometers, accounting for 65.71% of the city’s total area,^1^ providing an appropriate environment for the virus-mosquito-host cycle. Therefore, more surveillance and experimental transmission studies should be carried out in this region to alert the import and export of unknown viruses and their threat to animal and human health.

TAHV has been detected from more than one mosquito species worldwide, suggesting that TAHV PJ01 strain can be transmitted *via* a broad range of mosquito species. The urgent necessity is to determine the unknown routes of virus transmission. This study provides the first evaluation of vector competence and shows that *Ae. albopictus* mosquitoes are effective vectors for TAHV PJ01 strain, while *Cx. pipiens pallens* mosquitoes gradually cleaned the virus from the body. This disparity in vector competence between the two mosquito species may be conferred by the differences in innate immunity and epigenetics etc. (Houe et al., 2019). After oral infection, *Ae. albopictus* mosquitoes are highly susceptible to the TAHV PJ01 strain and had a fluctuating increase in IR, TR, DR and DE from 4 dpe to 18 dpe. At 2 dpe, 16.7 and 13.3% of mosquitoes showed salivary gland and saliva positive for TAHV PJ01 strain, respectively. This implies that TAHV PJ01 strain is able to break the midgut barrier and salivary gland barrier of *Ae. albopictus*, with an extrinsic incubation period (EIP) of 2 days. The EIP refers to the time required for the virus to reach the mosquito saliva after ingesting an infectious blood meal, and is influenced by diverse factors, such as mosquito species and population, virus genetic characteristics, virus-mosquito interactions, and environmental factors (Kramer and Ebel, 2003; Moudy et al., 2007). Previous studies showed that, for this geographic strain of *Ae. albopictus*, ZIKV SZ01 strain has an EIP of 6 days (Guo et al., 2020), and DENV2-FJ10, -FJ11 and − 43 were detected in salivary glands 7 days after ingestion (Guo et al., 2013, 2016). The fact that the TAHV PJ01 strain has such a short EIP of 2 days may be due to the genetic characteristics of the virus and the special interaction with the vector, which needs to be further explored. The duration of EIP is of great epidemiological significance in the prevention of arboviral diseases. In detail, *Ae. albopictus* typically ingest blood 2–3 times in a gonotrophic cycle (Delatte et al., 2009), and a shorter EIP can increase the possibility of virus transmission from mosquitoes to the bitten animal or human. Moreover, the transmission of TAHV PJ01 strain was further confirmed by the infection of suckling mice when they were bitten by virus-positive *Ae. albopictus*. TAHV could cause acute disease in immunocompetent suckling mice. 33.3% of the infected suckling mice showed neurological illness and death at day 5, at which time the virus titer in their brains reached 1.70 × 10^5^ PFU/mL (1.72 × 10^5^ PFU/g). However, 66.7% of the infected suckling mice showed no obvious disease symptoms. This interindividual variation in disease progression may be due to differences in initial viral acquisition *via* biting and immunity of the suckling mice. In brief, the shorter EIP of TAHV PJ01 strain and its pathogenicity and lethality in mammals force us to pay more attention to its emergence.

Transovarial transmission is considered to be the main route by which some arboviruses can maintain and complete their transmission cycle despite ecological constraints (Hardy et al., 1983). In previous researches, several viruses of the CSG, such as the LACV (Pantuwatana et al., 1974; Patrican et al., 1985), CEV (Reisen et al., 1990), JCV (Boromisa and Grimstad, 1986; Heard et al., 1990; Kramer et al., 1993), TVTV (Andrews et al., 1977; Christensen et al., 1978), SSHV (Schopen et al., 1991), San Angelo virus (Tesh, 1980; Tesh and Shroyer, 1980; Tesh and Cornet, 1981) and KEYV (JW et al., 1975), have been demonstrated transovarial transmission in diverse *Aedes* and *Culiseta* mosquito species *via* field observation and/or laboratory infection experiments. TAHV was also confirmed to be capable of transovarial transmission in *Culiseta annulata* (Bardos et al., 1975), *Ae. vexans* (Danielova and Ryba, 1979) and *Aedes aegypti* (Labuda et al., 1983). This is the first study to demonstrate transovarial transmission of TAHV (PJ01 strain) in *Ae. albopictus* mosquitoes. Moreover, TAHV can be detected in the first gonotrophic cycle after feeding on virus-infected blood meals, which indicated that the virus was already established in the germline tissues before ovarian maturation. At 2 dpe, TAHV PJ01 strain can be detected in 76.6% of the ovaries of blood-engorged female mosquitoes (Figure 2A), however, most ovaries were still immature at this very moment. Such rapid ovarian infection may be due to the rapid viral breach of midgut infection and release barrier, or due to the occurrence of midgut leakage, although the molecular mechanisms need to be further explored. With the ovaries development to 6 dpe, the virus-positive rate of ovaries reached a peak of 90.0%, and the virus load also reached a maximum of 5.91 ± 0.30 lg RNA copies/μl. From egg to the adult stage, IR, MIR and RNA copy showed downward trends both in the first and second gonotrophic cycle. On the one hand, this suggests that there may be important viral clearance mechanisms and innate immune changes during mosquito development. On the other hand, whether the decline of hatching, pupation and emergence rate of virus-infected eggs, larvae and adults is responsible for this result needs to be further proved. Moreover, the MIR values achieved for egg/larvae/adult were 3.2%/3.0%/1.3% in the first gonotrophic cycle and 3.1%/2.0%/1.3% in the second gonotrophic cycle, respectively. These high MIR values and high vRNA copies in the parental ovaries imply that the virus was efficiently maintained in nature by vertical transmission. In a word, the efficient vertical transmission of TAHV PJ01 strain is another important factor that we must pay more attention to.

In summary, this study characterized a novel virus, TAHV PJ01 strain, to which the *Ae. albopictus* population in China is susceptible and competent for viral replication and horizontal transmission. Moreover, the ability of transovarial transmission of *Ae. albopictus* suggests that this species can be a persistent survival host of TAHV PJ01 strain during unfavorable interepidemic periods and also a transportable reservoir of infection. Due to the lack of vaccines and targeted treatments, the prevention and control of arbovirus diseases relies heavily on vector control. However, with increased transport activities and continued climate warming, the geographical range of vector mosquitoes has expanded and crossed, resulting in more rapid and widespread transmission and continued evolution of the virus. Thus, the susceptibility of common mosquito species must be assessed to design and improve the comprehensive vector control strategy for managing viral infections. This study is an important complement for understanding the maintenance and transmission of *Orthobunyavirus* through mosquitoes, which can be used to predict and mitigate the risk of potential virus outbreaks in the future and has important epidemiological significance. Additionally, more adult mosquito control measures should be adopted to control mosquitoes if a TAHV PJ01 strain-related epidemic occurs.

## Data availability statement

The original contributions presented in the study are included in the article/Supplementary materials, further inquiries can be directed to the corresponding authors.

## Ethics statement

The animal study was reviewed and approved by Institutional Animal Care and Use Committee of the State Key Laboratory of Pathogen and Biosecurity, Beijing Institute of Microbiology and Epidemiology.

## Author contributions

TC, YJ, XG, and TZ designed the study. RL, XC, and FS isolated the virus. YJ contributed to deep sequencing and phylogenetic analysis. TC, NJ, and XJ contributed to oral infection and mosquitoes processing. TC contributed to mosquito rearing, horizontal transmission experiments, vertical transmission experiments, virus detection, vector competence analysis, and statistical analysis. TC and YJ wrote the manuscript. All authors reviewed, revised, and approved the final manuscript.

## Funding

This work was supported by the Infective Diseases Prevention and Cure Project of China under grant (no. 2017ZX1030404).

## Conflict of interest

The authors declare that the research was conducted in the absence of any commercial or financial relationships that could be construed as a potential conflict of interest.

## Publisher’s note

All claims expressed in this article are solely those of the authors and do not necessarily represent those of their affiliated organizations, or those of the publisher, the editors and the reviewers. Any product that may be evaluated in this article, or claim that may be made by its manufacturer, is not guaranteed or endorsed by the publisher.

## Supplementary material

The Supplementary material for this article can be found online at: https://www.frontiersin.org/articles/10.3389/fmicb.2023.1159835/full#supplementary-material

## Footnotes

^1^ https://www.panjin.gov.cn/html/1774/
