# Supplementary material for: Vector competence evaluation of mosquitoes for Tahyna virus PJ01 strain, a new Orthobunyavirus in China
Source: Front Microbiol. 2023 Apr 20;14:1159835. doi: 10.3389/fmicb.2023.1159835 (PMC10157254; doi:10.3389/fmicb.2023.1159835)
Supplement: Supplementary file 1 [file Table_1.DOCX]

Supplementary Material

Vector Competence Evaluation of Mosquitoes for Tahyna virus PJ01 strain, a New *Orthobunyavirus* in China

Tong Cai^1†^, Ran Liu^1†^, Yuting Jiang^1†^, Nan Jia^1^, Xianyi Jian^1^, Xiaolan Cheng^2^, Fenglin Song^2*^, Xiaoxia Guo^1*^ and Tongyan Zhao^1*^

*** Correspondence:** Fenglin Song: [songfl99@sina.com](mailto:songfl99@sina.com); Xiaoxia Guo: [guoxx99@163.com](mailto:guoxx99@163.com); Tongyan Zhao: [tongyanzhao@126.com](mailto:tongyanzhao@126.com).

# Supplementary Table 1

Supplementary Table 1. Primers and probe used in this study.

| Amplicons | Primer | Sequence (5’→3’) |
| --- | --- | --- |
| PCR | S1-F | CCGGTGCAAATGGATTTGATCCTG |
|  | S1-R | CCACCCACCCATTTAGCTGCTATA |
|  | M1-F | CAGGAAGTGGTTAGTCTCAGACTG |
|  | M1-R | CAAAGCTCCTGCTATGTCCTCA |
|  | M2-F | GGAATTGCACAGGGCCATTCTT |
|  | M2-R | CCGCAGTTTCTGTGCCTTCTATTG |
|  | M3-F | TTGCACATGGCAAGTTAGCCAC |
|  | M3-R | CCTTCATCCCTAACCCTACACATCC |
|  | L1-F | TGAGACAGACCCTCTAACAGTG |
|  | L1-R | CTGCAGAAGGGAATACTATGGC |
|  | L2-F | CCCAGCGGCTTAAAAAATGAC |
|  | L2-R | CACATTTTGGCCTCATACTCCC |
|  | L3-F | GAGGCCATGCCTAATTACACAG |
|  | L3-R | TCCAGCTTCTAACCCTACAGTG |
|  | L4-F | TAAAGCATGGATGCCCACCAAG |
|  | L4-R | ATGCCCACCTGACAGTATTTGC |
|  | L5-F | GGTGTTCATACTTCCAGCGAAAGC |
|  | L5-R | CGAACTCCCTATACATCACCCAGT |
| RACE | S-5’GSP | GATTACGCCAAGCTTCACCGGCTGTCAGGATACTTGGCTAGATG |
|  | S-3’GSP | GATTACGCCAAGCTTAACCGTCTTCTGCGACATCCACTTGTCC |
|  | M-5’GSP | GATTACGCCAAGCTTGGCTAGAAGAAGAAGGGGGCTCAGTCACTG |
|  | M-3’GSP | GATTACGCCAAGCTTAGGCCGGATGCTCTGTGCAATTTCAT |
|  | L-5’GSP | GATTACGCCAAGCTTCTCCAAGCGTGGTGAAAGCCACATGA |
|  | L-3’GSP | GATTACGCCAAGCTTCTCCTCAAAGAGTCTCCTTTCAGGGACTGGC |
| RT-qPCR | PJ-F | GCAAATGGATTTGATCCTGATGCAG |
|  | PJ-R | TTGTTCCTGTTTCCAGGAAAATGAT |
|  | PJ-P | FAM-TTCCTTAATGCCGCAAAAGCCAAGGCT-BHQ1 |

# Supplementary Table 2

Supplementary Table 2. The background information for viruses used for phylogenetic analyses in this study.

| Serogroup | Viruses | Abbreviation | Accession No. | | |
| --- | --- | --- | --- | --- | --- |
|  |  |  | L | M | S |
| California | California encephalitis virus | CEV | NC_055119 | NC_055118 | NC_055117 |
|  | La Crosse virus | LACV | NC_004108 | NC_004109 | NC_004110 |
|  | Snowshoe hare virus | SSHV | NC_055196 | NC_055197 | NC_055198 |
|  | Jamestown Canyon virus | JCV | NC_043559 | NC_043560 | NC_043558 |
|  | Inkoo virus | INKV | KT288284 | KT288285 | KT288286 |
|  | Chatanga virus | CHATV | KT288305 | KT288306 | KT288307 |
|  | Keystone virus | KEYV | NC_043629 | NC_043627 | NC_043628 |
|  | Jerry Slough virus | JSV | KX817318 | KX817319 | KX817320 |
|  | Lumbo virus | LUMV | NC_043632 | NC_043630 | NC_043631 |
|  | Melao virus | MELV | NC_043634 | NC_043633 | NC_043635 |
|  | San Angelo virus | SAV | NC_043637 | NC_043638 | NC_043636 |
|  | Serra do Navio virus | SDNV | NC_043641 | NC_043639 | NC_043640 |
|  | South River virus | SORV | KX817336 | KX817337 | KX817338 |
|  | Trivittatus virus | TVTV | KR149249 | KR149248 | KR149247 |
|  | Morro Bay virus | MBV | NA | NA | U31989 |
|  | Achiote virus | ACHOV | KY555808 | KY555809 | KY555810 |
|  | Infirmatus virus | INFV | KY569262 | KY569263 | KY569264 |
|  | Tahyna virus Prototype 92 Bardos | TAHV | HM036208 | HM036209 | HM036210 |
|  | Tahyna virus XJ0625 strain |  | NC_055207 | NC_055205 | NC_055206 |
|  | Tahyna virus XJ0708 strain |  | HM243137 | HM243138 | HM243139 |
|  | Tahyna virus XJ0710 strain |  | HM243140 | HM243141 | HM243142 |
| Bunyamwera | Bunyamwera virus | BUNV | NC_001925 | NC_001926 | NC_001927 |

# Supplementary Table 3

Supplementary Table 3. Infection, transmission and dissemination of TAHV PJ01 strain in *Ae. albopictus* and *Cx. pipiens pallens* after receiving virus-infected blood meals.

| Dpe | *Ae. Albopictus* | | | |  | *Cx. pipiens pallenss* | | | |
| --- | --- | --- | --- | --- | --- | --- | --- | --- | --- |
|  | IR | TR | DR | DE |  | IR | TR | DR | DE |
| 2 | 90.0% | 18.5% | 80.0% | 13.3% |  | 20.0% | 16.7% | 0.0% | 0.0% |
| 4 | 56.7% | 29.4% | 0.0% | 0.0% |  | 10.0% | 33.3% | 0.0% | 0.0% |
| 6 | 96.7% | 55.2% | 12.5% | 6.7% |  | 6.7% | 0.0% | - | 0.0% |
| 8 | 76.7% | 43.5% | 0.0% | 0.0% |  | 3.3% | 0.0% | - | 0.0% |
| 10 | 70.0% | 76.2% | 12.5% | 6.7% |  | 3.3% | 100.0% | 0.0% | 0.0% |
| 12 | 80.0% | 66.7% | 25.0% | 13.3% |  | 6.7% | 50.0% | 0.0% | 0.0% |
| 14 | 80.0% | 62.5% | 26.7% | 13.3% |  | 3.3% | 0.0% | - | 0.0% |
| 16 | 93.3% | 64.3% | 16.7% | 10.0% |  | 3.3% | 0.0% | - | 0.0% |
| 18 | 73.3% | 81.8% | 50.0% | 30.0% |  | / | / | / | / |

30 mosquitoes were dissected in each sampling day.

IR (Infection rate) = number of mosquitoes with infectious midgut / total number of mosquitoes tested.

TR (Transmission rate) = number of mosquitoes with infectious salivary gland / number of mosquitoes with infectious midgut.

DR (Dissemination rate) = number of mosquitoes with infectious saliva / number of mosquitoes with infectious salivary gland.

DE (Dissemination efficiency) = number of mosquitoes with infectious saliva / total number of mosquitoes tested.
